# 

**Published:** 1893-03-11

**Authors:** 


OUR NEW OFFICES.

428, Strand, W.C., will henceforth be the Office of this Journal.

### NOTICE TO CORRESPONDENTS.

All MS., Letters, Books for Review, and other matters intended for the Editor should be addressed The Editor, The Lodge, Porchester Square, London, W.

The Editor cannot undertake to return rejected M.S., even when accompanied by stamped directed envelope.

**Notice to Advertisers and Subscribers.**

All Advertisements, Orders for Copies of The Hospital, and Business Communications should be addressed to The Publisher, *not to the Editor*, The Hospital, 428, Strand, W.C.

The Cost of Subscription, post free, is as follows:—Per week, 2½d.; per quarter, 2s. 6d.; per half-year, 5s.; per annum, 8s. 6d. Foreign:—Per annum, 10s. 8d.; payable, in all cases, in advance.

**"Burdett's Hospital Annual," 1893.**

Fourth year of publication. 700 pp. Boards, gilt lettering. A most complete guide to British, Colonial, Indian, and American Hospitals, Dispensaries, Nursing and Convalescent Institutions, and Asylums. Price 5s. Published by The Scientific Press (Limited), 428, Strand, W.C.

**NOTICE.—The Index for Vol. XII. (ending September 1892) is on sale at the Office of this Journal, price 2½d., post free.**

### Scraps and Gleanings.

The benefit of salipyrine in the cure of neuralgia is more decided than is the use of antipyrin or the salicylates, and there is less risk as to its after effects.

The Woodlands Convalescent Home, an excellent institution in connection with the Bradford Infirmary, shows a balance to the good of £342 5s. 5d. this year.

A shelter on the banks of the Thames has been in operation for some time for the lodging of London homeless poor. It is erected by the Salvation Army, and the charge is 1d. the night.

The House Committee of the Montrose Infirmary report that the air space allowed for each patient in the wards is quite insufficient; it is considered therefore necessary to extend the building.

The Lord Mayor has addressed a letter to all mayors of the United Kingdom urging them to see that there are nurses specially trained for cholera in the event of an outbreak of that disease.

The *Dublin Express* states that an order has been issued by the Lord Lieutenant and Privy Council prohibiting the importation of firearms and ammunition into Ireland except under specialised conditions.

Mr. Barringer, of Mansfield, has purchased a plot of land near the District Hospital, and has raised £45 towards the cost, which was originally £125. The subscription list of Mansfield Hospital is a satisfactory one this year.

A heavy debt of £4,000 hangs over the Bradford Infirmary for Sick Children. Mr. Agnew, who is a liberal supporter of the institution, offers £500 towards the debt-clearing purse, on condition of the forthcoming of the rest of the money.

For Student of Medicine and Medical Appointments see pages X. and XI. overleaf.

### The Student of Medicine.

[All communications for this Supplement should be addressed to The Editor of The Hospital, at 428, Strand, with the words, "Students' Column," written in the left hand top corner of the envelope.]

### APPOINTMENTS.

R. D. Ashby, L.D.S., appointed Consulting Dental Surgeon to the Royal Northern Sea-Bathing Infirmary, Scarborough. W. M. Barclay, F.R.C.S.Eng., appointed Surgeon to the Bristol General Hospital. V. E. Barrow, L.R.C.P., L.R.C.S., L.F.P.S. Glasg., appointed Medical Officer for the Uffculme District of the Tiverton Union. M. R. J. Behrendt, L.R.C.P., L.R.C.S. Edin., reappointed Medical Officer for the Scunthorpe Urban Sanitary District. A. O. Bentham, L.R.C.P.Lond., M.R.C.S. Eng., appointed Medical Officer for the Abram District of the Wigan Union. A. E. Berry, M.B.Lond., M.R.C.S., L.R.C.P., appointed Resident Medical Officer to the Monsall Fever Hospital, Manchester. G. W. H. Bird, B.A., Cantab., L.R.C.P., M.R.C.S., appointed Clinical Assistant in the Special Department for Diseases of the Skin, St. Thomas's Hospital. W. A. Bowring, L.R.C.P., M.R.C.S., appointed Junior Obstetric House-Physician to St. Thomas's Hospital. T. Brayton, L.R.C.P., L.R.C.S.Edin., appointed Medical Officer for the Hindley District of the Wigan Union. W. F. Clark, L.R.C.P., L.R.C.S.I., reappointed Medical Officer of Health for Cheshunt. T. E. Constant, L.R.C.P.Lond., M.R.C.S., L.D.S., appointed Honorary Consulting Dental Surgeon to the Royal Northern Sea Bathing Infirmary, Scarborough. R. T. E. B. Cooke, F.R.C.S., L.R.C.P.Edin., M.R.C.S., appointed Consulting Medical Officer to the Royal Northern Sea-Bathing Infirmary, Scarborough. R. Cuff, M.B.Lond., M.R.C.S., appointed Medical Officer to the Royal Northern Sea-Bathing Infirmary, Scarborough. F. Day, W.H.L., L.R.C.P.Lond., M.R.C.S.Eng., appointed Medical Officer for the Fifth District of the Hitchin Union. M. R. P. Dorman, M.A., M.B., B.C.Cantab, L.R.C.P., M.R.C.S., appointed Non-Resident House-Physician to St. Thomas's Hospital. D. Drew, M.D., B.S.Lond., F.R.C.S.Eng., appointed House-Surgeon to the North Staffordshire Infirmary, Stoke-on-Trent. G. F. Easton, M.D., L.R.C.S.Edin., appointed Medical Officer of Health to the Alnwick Local Board. R. Eatough, M.D.Aber., appointed District Medical Officer of the Township of Saddleworth. W. R. Edmond, M.B., C.M., L.R.C.P.Edin., M.R.C.S.Eng., appointed Medical Officer for the Chew Magna District of the Clutton Union. R. K. Ellis, M.B., B.Ch.Oxon, appointed Senior Obstetric House Physician to St. Thomas's Hospital. N. H. Forbers, L.R.C.P. Lond., M.R.C.S.Eng., appointed Honorary Surgeon to the Tunbridge Wells Fire Brigade. H. J. Frederick, L.R.C.P., M.R.C.S., L.S.A., appointed Clinical Assistant in the Special Department for Diseases of the Throat, St. Thomas's Hospital. E. F. Fussell, M.B.Aberd., M.R.C.P.Lond., reappointed Medical Officer of Health to the Newhaven Local Board. G. J. Gibson, M.D.Q.U.I., appointed Court Surgeon to the Totnes Branch of the Rational Sick and Burial Society. W. Gordon, M.B., B.C.Cantab., M.R.C.P.Lond., Physician to the Devon and Exeter Hospital, appointed Physician to the Exeter Dispensary. L. D. Gover, L.R.C.P., M.R.C.S., appointed Clinical Assistant in the Special Department for Diseases of the Ear, St. Thomas's Hospital. W. R. Gowers, M.D.,Lond., F.R.C.P., M.R.C.S., appointed Extra Physician to the National Hospital for the Paralyzed and Epileptic, Queen Square, Bloomsbury. G. B. Griffiths, L.R.C.P. Lond., M.R.C.S., appointed Medical Officer for the Stoke-upon-Trent District of the Southwell Union. E. M. Hainworth, L.R.C.P., M.R.C.S., reappointed Non-Resident House-Physician to St. Thomas's Hospital. C. H. Hallett, M.B., C.M.Glasg., appointed Medical Officer for the Bradninch District of the Tiverton Union. J. M. Heron,

### MEDICAL APPOINTMENTS.

Salary £100 per annum, with Board, Apartments, and Washing.

91, West Regent Street, Glasgow. (609)

THE STUDENT OF MEDICINE—(*continued*).

M.B., B.Ch., appointed Assistant House-Surgeon to the East Suffolk Hospital, Ipswich. J. W. Hicks, L.R.C.P., M.R.C.S., reappointed Clinical Assistant in the Special Department for Diseases of the Throat, St. Thomas's Hospital. J. Hodges, M.R.C.S.Eng., L.S.A., appointed Assistant Medical Officer to the Suffolk General Hospital. F. H. G. Hutchinson, M.B., C.M., appointed Junior House Surgeon to the Cheltenham General Hospital, *vice* G. H. Prance, M.B., C.M., resigned. C. S. Jeffé, L.R.C.P., M.R.C.S., reappointed Resident House-Physician to St. Thomas's Hospital. D. O. Kerr, M.B., C.M. Edin., reappointed Medical Officer for the Keelby District of the Grimsby Union. J. Kilner, F.R.C.S.Eng., appointed Consulting Medical Officer to the Suffolk General Hospital. R. R. Law, B.A., M.B., B.C.Cantab, appointed House-Surgeon to St. Thomas's Hospital.

### VACANCIES.

The following vacancies are announced this week, the amount of salary in each case being given after the name of the institution:

### Resident Medical Officers.

Cancer Hospital (Free), Fulham Road, S.W. House Surgeon. Appointment for six months. Salary at the rate of £50 per annum, with board and residence. Applications to the Secretary by March 18th.

County Lunatic Asylum, Whittingham, Lancashire. Junior Assistant Medical Officer. Salary £100 per annum, with apartments, board, and washing. Applications to the Superintendent.

General Hospital, Birmingham. House Physician. Salary £70 per annum, with residence, board, and washing. Applications to Howard J. Collins, House Governor, by March 25th.

York County Hospital, York.—Senior House Surgeon, doubly qualified. Salary £100 per annum, with board, apartments, and washing. Applications to C. E. Pinfold, Secretary, by March 10th.

Liverpool Infirmary for Children, Myrtle Street, Liverpool. House Surgeon. Salary £85 per annum, with board and lodging. Applications to C. W. Carver, Honorary Secretary, by March 18th.

St. Marylebone General Dispensary, 77, Welbeck Street, Cavendish Square, W. Obstetric Physician. Applications to Secretary by March 8th.

Manchester Royal Infirmary. Assistant Medical Officer at the Monsall Fever Hospital, unmarried. Appointment for twelve months. Salary £100 per annum, with board and residence. Applications to the Chairman of the Board, Royal Infirmary, Manchester, by March 21st.

St. Peter's Hospital for Stone and Urinary Diseases, Henrietta Street, Covent Garden, W.C. House Surgeon. Appointment for six months. Honorarium 25 guineas, board, lodging, and washing. Applications to Walter E. Scott, Secretary, by March 21st.

### Non-Resident Medical Officers.

Bristol Dispensary, Castle Green, Bristol. Vacancy in the Medical Staff, double qualification. Applications to Edward Stock, Secretary, by March 10th.

Bedford General Dispensary.—Dispenser, under 45 years of age. Salary £70 per annum. Applications to the Secretary by March 22nd.

Board of Works for the Poplar District.—Medical Officer of Health for the Poplar and Bromley Parishes. Salary £400 per annum. Applications, endorsed "Appointment of Medical Officer," to be sent to the Offices of the Board, 117, High Street, Poplar, by March 15th.

Dunfanaghy Union.—Dunfanaghy Dispensary. Medical Officer. Salary £100 per annum, with £10 as Medical Officer of Health, and registration and vaccination fees. Applications to Thomas A. Ingram, Honorary Secretary, by March 11th Election on March 18th.

Dunfanaghy Union.—Medical Officer for Workhouse and Fever Hospital. Salary £30 per annum.

Farringdon General Dispensary, Bartlett's Buildings, Holborn Circus, E.C.—Surgeon. Applications to W. K. Taunton, Honorary Secretary, by March 11th.

Guy's Hospital, S.E. Assistant Dental Surgeon. Applications to the Dean by March 4th.

Italian Hospital, Queen's Square, Bloomsbury, W.C. Honorary Physician. Applications to the Honorary Secretary, Arthur Serena, 8, Austin Friars, E.C.

Parish of Glenelg. Medical Officer for the Northern Division. Salary £95 per annum, with free house and gardens. Applications to D. McLure, Inspector of the Poor, Glenelg, by March 15th.

University of Glasgow. Four Examinerships for Degrees in Medicine. Annual fee £30. Applications to Alan E. Clapperton, Secretary, to the Court, 91, West Regent Street, Glasgow, by March 11th.

Yorkshire College, Leeds. Demonstrator in Anatomy. Applications to the Registrar.

